# Safety and Efficacy of Nerandomilast in Patients With Pulmonary Fibrosis: A Systematic Review and Meta‐Analysis of Randomized Controlled Trials

**DOI:** 10.1111/crj.70181

**Published:** 2026-03-11

**Authors:** Humna Shahzad, Usama Afzaal, Fahad Saleem, Ahmad Hassan Gul, Freya Thummar, Hafiz Nadir Murtaza, Abel Gelan, Maria Ahsan, Rafay Irfan, Ahmad Nawaz, Uzair Jafar, Asma'a Munasar Ali Alsubari, Muhammad Ehsan, Ahmed Nadeem, Praveen Kumar Komminni, Juan Iribarren

**Affiliations:** ^1^ Department of Medicine Nishtar Medical University Multan Pakistan; ^2^ Department of Internal Medicine Baptist Hospitals of Southeast Texas Beaumont Texas USA; ^3^ Department of Medicine Punjab Medical College Faisalabad Pakistan; ^4^ Department of Medicine C. U. Shah Medical College Surendranagar India; ^5^ Department of Medicine King Edward Medical University Lahore Pakistan; ^6^ Department of Public Health Tulane University Louisiana USA; ^7^ Department of Medicine CMH Kharian Medical College Kharian Pakistan; ^8^ Faculty of Medicine Sana'a University Sana'a Yemen; ^9^ Department of Internal Medicine Cleveland Clinic Cleveland Ohio USA; ^10^ Department of Internal Medicine Jacobi Medical Center/North Central Bronx Hospital New York New York USA

**Keywords:** IPF, meta‐analysis, nerandomilast, PDE4 inhibitors, pulmonary fibrosis

## Abstract

**Objective:**

Nerandomilast, an oral phosphodiesterase‐4 (PDE4) inhibitor, has shown potential in slowing the progression of pulmonary fibrosis. This meta‐analysis evaluated the efficacy and safety of nerandomilast in preserving lung function among patients with pulmonary fibrosis.

**Methods:**

MEDLINE, Embase, the Cochrane Library, and ClinicalTrials.gov were systematically searched for randomized controlled trials (RCTs) comparing nerandomilast with placebo. Study quality was assessed using the Cochrane Risk of Bias 2.0 tool. Analyses were performed in RevMan 5.4 using random‐effects models with risk ratios (RR) and mean differences (MD) as effect measures.

**Results:**

Four RCTs (*n* = 2515) were included. Nerandomilast significantly attenuated the decline in forced vital capacity (FVC) compared with placebo (MD: 69.25 mL, 95% CI: 52.1–86.29), but did not improve diffusing capacity for carbon monoxide (DLCO) (MD: 0.84, 95% CI: −0.56 to 2.24). It was associated with a lower pooled risk of all‐cause mortality (RR: 0.68, 95% CI: 0.52–0.88) without increasing adverse events (RR: 1.00, 95% CI: 0.98–1.02) or serious adverse events (RR: 0.93, 95% CI: 0.76–1.14).

**Conclusion:**

Nerandomilast appears to slow lung function decline in pulmonary fibrosis without added safety risks. Although a lower pooled risk of mortality was observed, individual trials were not powered for mortality outcomes, and event rates were low; therefore, this finding should be interpreted cautiously. Given the heterogeneity of pulmonary fibrosis phenotypes and trial designs, further large‐scale RCTs should explore standardized outcomes, subgroup effects, and combination strategies with nintedanib or pirfenidone.

## Graphical abstract

Nerandomilast appears to slow lung function decline and reduce mortality in pulmonary fibrosis without added safety risks. It may represent a valuable adjunct to existing antifibrotics or a standalone therapy for patients intolerant to standard treatment.

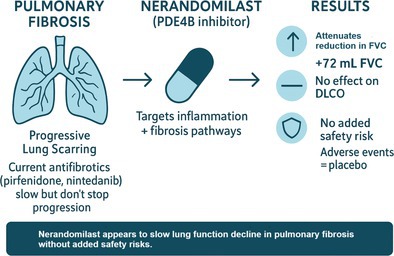

## 1 Introduction

Pulmonary fibrosis (PF) is a progressive interstitial lung disease (ILD) characterized by lung scarring caused by excessive extracellular matrix (ECM) accumulation and uncontrolled fibroblast growth [1]. In the United States, ILD affects about 650 000 individuals and causes 25 000 to 30 000 deaths annually. Idiopathic pulmonary fibrosis (IPF) is the most common subtype, representing nearly one‐third of all ILD cases, with a median survival of 3–5 years after diagnosis [2]. Other fibrotic lung diseases with similar progression are grouped as progressive pulmonary fibrosis (PPF) [3].

The clinical course of PF is variable, ranging from gradual decline to rapid deterioration; hence, early intervention is necessary. The American Thoracic Society and European Respiratory Society recommend both pirfenidone and nintedanib for the treatment of IPF, while nintedanib alone is recommended for PPF [4]. However, many patients continue to progress despite therapy, and adverse effects often limit treatment tolerability [5, 6, 7]. Both agents produce dose‐related gastrointestinal issues, and nintedanib, in particular, is associated with hepatic enzyme elevations.

Nerandomilast (BI 1015550) is a novel, orally administered, preferential inhibitor of phosphodiesterase 4B (PDE4B), in clinical development. It acts as an antifibrotic agent by modulating cAMP signaling pathways to suppress fibroblast proliferation and extracellular matrix deposition [8]. Early‐phase studies suggested stabilization of lung function. More recently, trials demonstrated significant reductions in forced vital capacity (FVC) decline in both IPF and non‐IPF PF, with an acceptable safety profile.

Previous reviews have shown that nintedanib and pirfenidone reduce the rate of lung function decline in PF, but evidence for nerandomilast has been limited to IPF and early trials. The only prior meta‐analysis including nerandomilast was restricted to early‐phase IPF data, excluding broader PF populations and outcomes such as diffusing capacity for carbon monoxide (DLCO) [9]. Since then, the FIBRONEER‐IPF and FIBRONEER‐ILD trials have confirmed that nerandomilast slows FVC decline in both IPF and non‐IPF PF, regardless of background antifibrotic therapy, and early studies have verified its safety [4, 10]. These results make nerandomilast the first oral antifibrotic in over a decade to slow lung function decline in PF [11]. We therefore conducted a meta‐analysis of randomized controlled trials (RCTs) to evaluate the safety and efficacy of nerandomilast in PF to provide an updated assessment of its clinical value.

## 2 Materials and Methods

This systematic review and meta‐analysis was performed according to the guidelines of the Cochrane Handbook for Systematic Reviews of Interventions and reported according to the Preferred Reporting Items for Systematic Reviews and Meta‐Analyses (PRISMA) statement (Table S1) [12, 13]. The study protocol was registered with the International Prospective Register of Systematic Reviews (PROSPERO) under the ID CRD420251144582.

### 2.1 Data Sources and Search Strategy

We performed a comprehensive search of several databases and trial registers from their inception up to July 2025, including Cochrane Central Register of Controlled Trials (CENTRAL), MEDLINE (via PubMed), Embase (via Ovid), and ClinicalTrials.gov, to retrieve RCTs evaluating the use of nerandomilast in patients with PF. Our search strategy employed a combination of relevant keywords and Medical Subject Headings (MeSH) terms. Additionally, a manual review of the reference lists of included studies was conducted to identify any additional relevant studies. The detailed search strategy is shown in Table S2.

### 2.2 Eligibility Criteria

The following inclusion criteria were used: (1) study design: RCTs; (2) population: adult patients (> 18 years of age) with idiopathic or PPF; (3) intervention: nerandomilast at any dose; (4) comparator: placebo; (5) outcomes: studies reporting at least one outcome of interest.

The exclusion criteria were as follows: (a) any study design other than RCTs, such as quasi‐randomized trials, observational studies, case reports, literature reviews, systematic reviews, and meta‐analyses; (b) animal studies; (c) studies missing clinical information relevant to the outcomes being investigated.

### 2.3 Study Selection

All the articles retrieved from the search were imported into the Rayyan tool. Two independent reviewers initially screened titles and abstracts for inclusion, and articles that did not satisfy the eligibility criteria were excluded. Full‐text manuscripts of the remaining eligible studies were retrieved and thoroughly examined for inclusion. Any disagreements between the reviewers were resolved by reaching a consensus or discussion with a third independent reviewer.

### 2.4 Data Extraction

Two authors independently performed the data extraction using a prepiloted Microsoft Excel spreadsheet. A third reviewer resolved any discrepancies. The data extracted from each eligible study included the author's name, publication year, country of origin, study design, total sample size in both groups, mean age, mean baseline FVC, mean baseline diffusion capacity of lung for carbon monoxide, percentage of the predicted value, time since diagnosis, antifibrotic treatment, and the reported outcomes of interest.

### 2.5 Quality Assessment

The Cochrane Collaboration tool for assessing Risk of Bias (RoB 2.0) was employed for analyzing bias risk in RCTs by evaluating the following domains [14]: (1) risk of bias arising from the randomization process; (2) effect of assignment to intervention; (3) missing outcome data; (4) risk of bias in measurement of outcome; and (5) risk of bias in selection of reported result. Each domain was categorized as “low risk,” “some concerns,” and “high risk” according to the predefined criteria.

### 2.6 Data Analysis

Data syntheses were performed using Review Manager (RevMan Version 5.4.1). The dichotomous variables were pooled as risk ratios with 95% CI, and continuous variables as mean with standard deviation. A random‐effects model was applied to all outcomes. Statistical heterogeneity was evaluated using Higgins' *I* ^2^ statistics. An *I* ^2^ < 30% indicated low heterogeneity, while 30%–70% was considered moderate, and > 70% indicated high heterogeneity. Publication bias was not evaluated through either visual inspection of funnel plots or statistical regression tests because of the lower statistical accuracy of those tests in meta‐analyses with fewer than 10 included studies. Statistical significance was defined as a *p*‐value of less than 0.05. A prespecified subgroup analysis stratified by disease subtype (IPF vs. PPF) for primary outcomes was conducted.

## 3 Results

**FIGURE 1:**
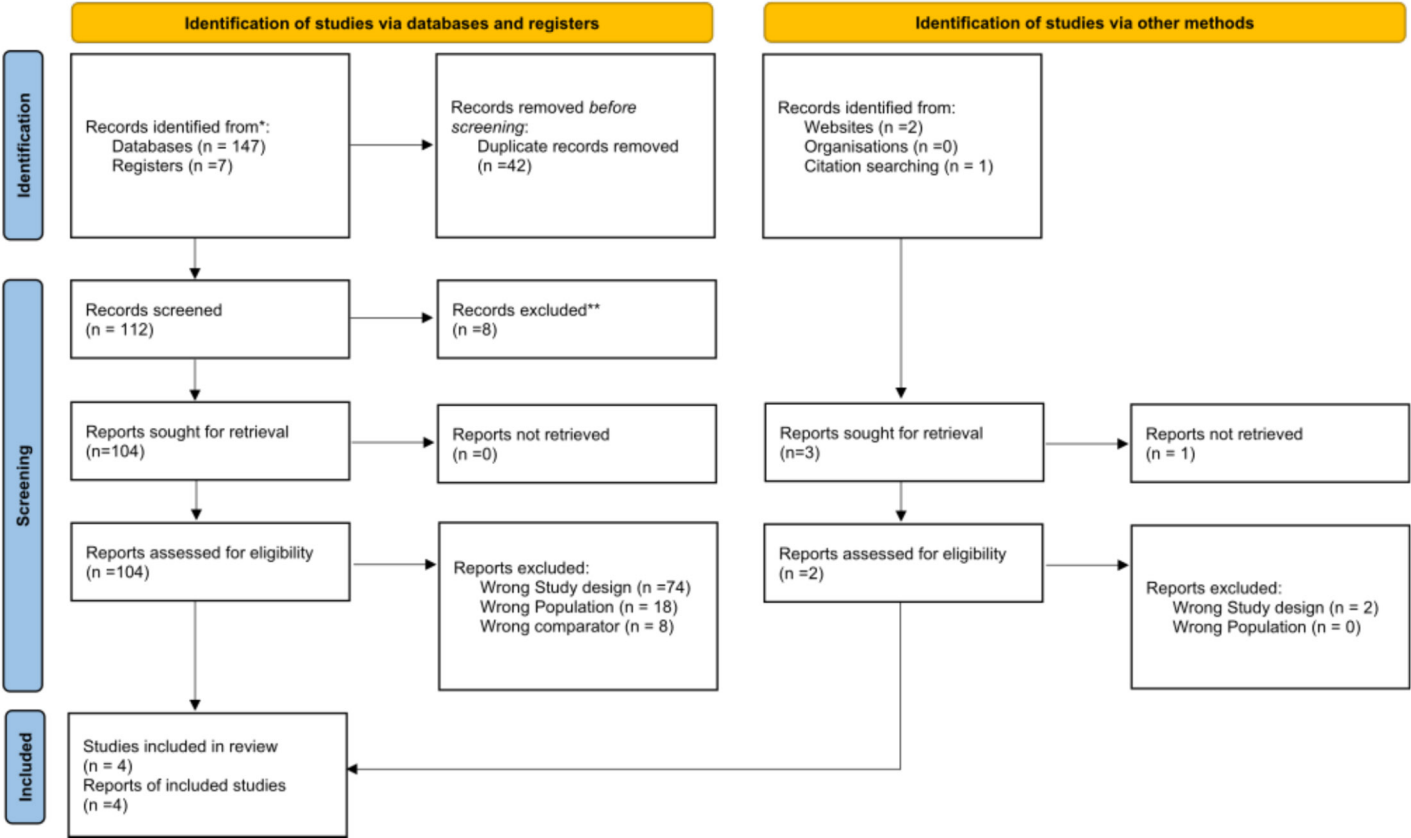
PRISMA flowchart for the screening process.

After going through an extensive screening process, four studies out of 68 fulfilled our inclusion criteria [4, 10, 15, 16]. The detailed screening process is illustrated in Figure 1. There were 2515 people in these RCTs across multiple countries, in which 1675 received nerandomilast therapy. Two were Phase III trials [4, 10], while the remaining two were Phase Ic and Phase II [15, 16]. The follow‐up period ranged from 4 to 54 weeks. There were two doses of nerandomilast (9 and 18 mg) in our two large trials [4, 10]. Three of the RCTs included patients with IPF, while Maher et al. [4] included patients with PPF. In all the studies, a placebo was used in the control group. The remaining baseline characteristics are presented in Table 1.

**TABLE 1:** Characteristics of the included studies.

Study ID	Study type	Location	Sample size	Study population	Dosage (intervention)	Follow‐up duration in weeks	Age in years (mean ± SD)	FVC in mL (mean ± SD)	Time since diagnosis of IPF in years (mean ± SD)	DLCO—percentage of predicted value (mean ± SD)	Antifibrotic treatment—no. (%)
**Richeldi 2025**	Phase 3, double‐blind, randomized controlled trial	36 countries	1177 (392 vs. 392 vs. 393)	Patients aged ≥ 40 years who had IPF confirmed by CT, an FVC of at least 45% of the predicted value, and a diffusing capacity of the lungs for carbon monoxide (DLCO) of at least 25% of the predicted value.	Nerandomilast 18 or 9 mg twice daily	52 weeks	70.3 ± 7.8 vs. 69.9 ± 7.5	2827 ± 758 vs. 2864 ± 805	3.6 ± 2.8 vs. 3.5 ± 2.7	51.5 ± 17.5 vs. 49.4 ± 15.8	305 (77.8%) vs. 306 (77.85%)
**Maher 2025**	Phase 3, double‐blind, randomized controlled trial	44 countries	1176 (391 vs. 393 vs. 392)	Patients aged ≥ 18 years with a diagnosis of non‐IPF interstitial lung disease (ILD) and > 10% fibrotic lung involvement on high‐resolution CT obtained within the past 12 months.	Nerandomilast 18 or 9 mg twice daily	52 weeks	66.0 ± 9.8 vs. 66.6 ± 10.3	2381 ± 723 vs. 2354 ± 766	4.6 ± 4.8 vs. 3.9 ± 3.6	49.4 ± 17.5 vs. 49.7 ± 16.5	—
**Richeldi 2022**	Phase 2, double‐blind, randomized controlled trial	22 countries	147 (97 vs. 50)	Patients aged ≥ 40 years who had a diagnosis of IPF confirmed by CT, FVC of at least 45% of the predicted value, and a diffusing capacity of the lungs for carbon monoxide (DLCO), between 25% and less than 80% of the predicted value.	Nerandomilast 18 mg twice daily	12 weeks	69.6 ± 7.46 vs. 69.65 ± 10.16	2829.73 ± 791.81 vs. 2777.45 ± 948.93	3.66 ± 3.25 vs. 3.05 ± 3.06	50.48 ± 17.5 vs. 47.75 ± 13.39	49 (50.5%) vs. 25 (50%)
**Maher 2022**	Phase 1c, double‐blind, randomized controlled trial	11 sites in seven European countries	15 (10 vs. 5)	Patients aged ≥ 40 years with an IPF diagnosis per international guidelines, without nintedanib or pirfenidone treatment within 30 days before enrolment or during the study period.	Nerandomilast 18 mg twice daily	4 to 12 weeks	69.5 ± 10.1 vs. 70.2 ± 3.3	3699.9 ± 1179.1 vs. 3322.2 ± 1024.6	—	61.6 ± 30.2 vs. 62.4 ± 18.8	—

### 3.1 Risk of Bias in Included Studies

**FIGURE 2:**
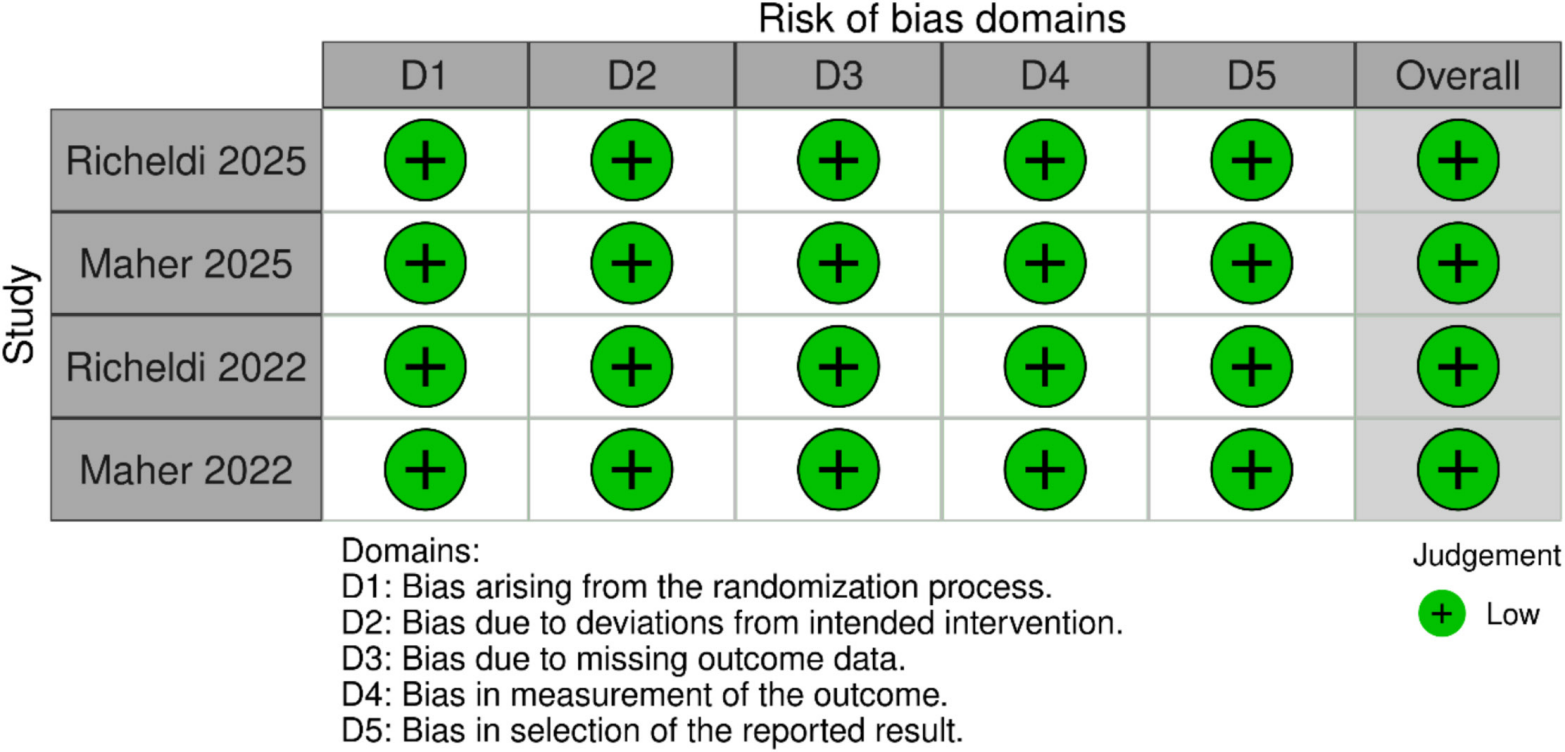
Risk of bias assessment of included studies.

The quality assessment of the included studies is presented in Figure 2. All four of the studies included in our meta‐analysis were at low risk of bias [4, 10, 15, 16].

### 3.2 Results of Meta‐Analysis

#### 3.2.1 Primary Outcomes

##### 3.2.1.1 Mean Change in FVC From Baseline

**FIGURE 3:**
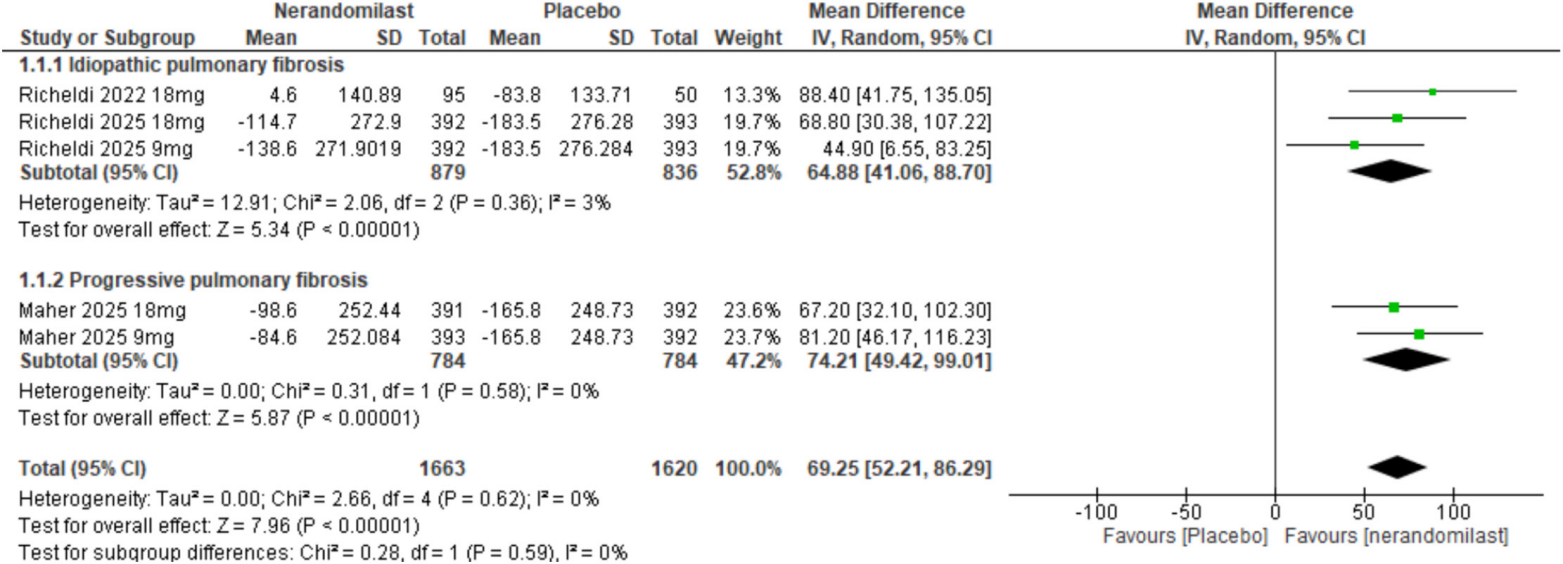
Effect of nerandomilast versus placebo on the mean change in FVC from baseline.

Three RCTs (*n* = 2498) evaluated the effect of nerandomilast on the change in FVC from baseline. Pooled analysis showed that nerandomilast was associated with a slower decline in FVC over 52 weeks as compared with control (MD: 69.25, 95% CI: 52.1–86.29, *p* < 0.00001; Figures 3 and S1). No significant heterogeneity was observed across studies (*I* ^2^ = 0%, *p* = 0.62). A subgroup analysis based on the type of ILD revealed no significant subgroup difference (*p* = 0.59).

##### 3.2.1.2 Change From Baseline in DLCO Percentage of the Predicted Value

**FIGURE 4:**
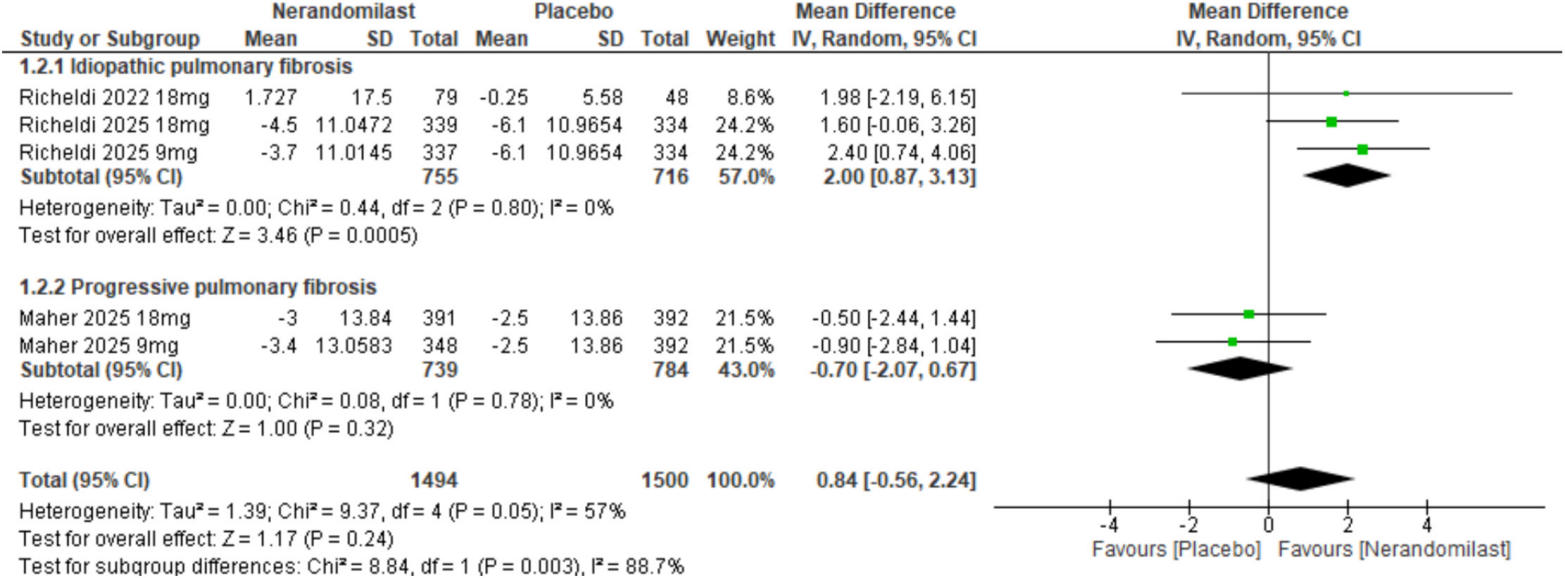
Effect of nerandomilast versus placebo on the change from baseline in DLCO percentage of the predicted value.

Three RCTs (*n* = 2268) assessed the effect of nerandomilast on the change in diffusing capacity of the lungs for carbon monoxide (DLCO) from baseline. The pooled analysis demonstrated no statistically significant difference between nerandomilast and control (MD: 0.84, 95% CI: −0.56 to 2.24, *p* = 0.19; Figures 4 and S2). The statistical heterogeneity was found to be moderate (*I* ^2^ = 57%, *p* = 0.05). A subgroup analysis by type of ILD showed a significant subgroup difference (*p* = 0.003).

#### 3.2.2 Secondary Outcomes

##### 3.2.2.1 All‐Cause Mortality

**FIGURE 5:**
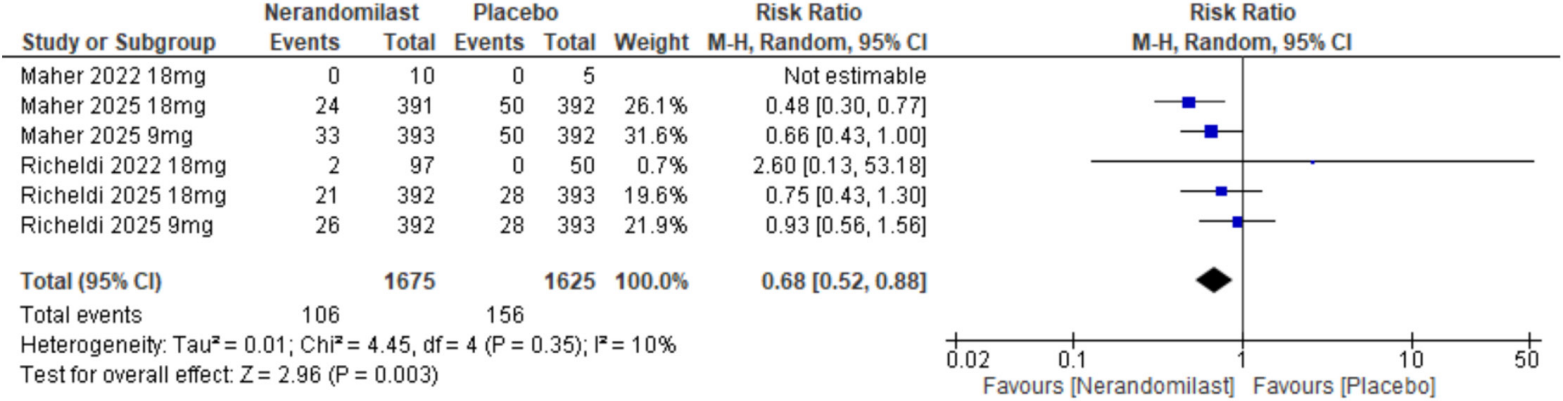
Effect of nerandomilast versus placebo on the risk of all‐cause mortality.

Four RCTs (*n* = 2515) evaluated the effect of nerandomilast on all‐cause mortality. The pooled analysis demonstrated that nerandomilast significantly reduced the risk of all‐cause mortality compared with control (RR: 0.68, 95% CI: 0.52–0.88, *p* = 0.003; Figures 5 and S3). Heterogeneity across studies was low (*I* ^2^ = 10%, *p* = 0.35).

##### 3.2.2.2 Risk of Adverse Events

Four RCTs (*n* = 2515) assessed the risk of adverse events with nerandomilast compared to the control group. The pooled analysis revealed no significant difference between the two groups (RR: 1.00, 95% CI: 0.98–1.02, *p* = 0.97; Figures S4 and S5). There was no evidence of heterogeneity across studies (*I* ^2^ = 0%, *p* = 0.81).

##### 3.2.2.3 Risk of Serious Adverse Events

Four RCTs (*n* = 2515) reported serious adverse events among patients treated with nerandomilast compared with the control. The pooled analysis showed no statistically significant difference between the groups (RR: 0.93, 95% CI: 0.76–1.14, *p* = 0.08; Figures S6 and S7). No heterogeneity was detected across studies (*I* ^2^ = 0%, *p* = 0.98).

##### 3.2.2.4 Adverse Events Leading to Treatment Discontinuation

Four RCTs (*n* = 2515) evaluated adverse events leading to treatment discontinuation. The pooled analysis showed no statistically significant difference between nerandomilast and control (RR: 1.01, 95% CI: 0.65–1.56, *p* = 0.98; Figures S8 and S9). Heterogeneity was substantial (*I* ^2^ = 83%, *p* = 0.0001).

## 4 Discussion

This systematic review and meta‐analysis comprising data from 2515 patients assesses the safety and efficacy of nerandomilast in PF. Our analysis showed that nerandomilast was associated with a statistically significant attenuation of FVC decline over time compared with placebo. However, no significant effect on the DLCO was observed. Importantly, nerandomilast treatment was linked to a lower pooled risk of all‐cause mortality compared with placebo without increasing the risk of adverse events, serious adverse events, or treatment discontinuation due to adverse events.

Preclinical studies and early clinical data support PDE4 inhibition as an adjunctive strategy to current antifibrotics, as PDE4 inhibitors reduced experimental lung fibrosis both as a monotherapy and add‐on therapy, although larger phase‐3 studies are required to confirm efficacy and fully characterize safety [17]. Our results are consistent with previous evidence on PDE4 inhibitors, while also highlighting two novel findings. First, this is the first meta‐analysis to pool randomized evidence specific to nerandomilast in both IPF and PPF. Second, unlike previous PDE4 inhibitors such as roflumilast and CHF6001, which have not demonstrated a survival benefit or reported mortality effects [18, 19], our analysis identified a significant reduction in all‐cause mortality, highlighting a potential advantage. A recent network meta‐analysis in IPF, which included seven RCTs, found the greatest FVC preservation with high‐dose nerandomilast plus nintedanib, with nintedanib monotherapy also performing well [9]. Our meta‐analysis, encompassing four RCTs in both IPF and PF‐ILD, similarly demonstrated significant FVC benefit, suggesting nerandomilast may be effective across a broader range of PF. Aligned with our safety results, both these studies reported no difference in adverse events.

The nonsignificant effect on DLCO warrants further consideration. One possible explanation is that the intervention primarily targets interstitial remodeling while DLCO reflects a composite of alveolar–capillary membrane integrity, pulmonary capillary blood volume, and hemoglobin concentration [20]. The relatively short follow‐up duration in early‐phase trials may also have been insufficient to capture meaningful improvements in diffusion capacity. In our analysis, the evidence primarily came from two large, multicentre phase III RCTs, FIBRONEER‐IPF, which investigated patients with IPF, and FIBRONEER‐ILD, which focused on patients with PPF. The remaining two studies were smaller phase II and phase Ic trials [4, 10, 15, 16]. This indicates that the population included in our meta‐analysis was diverse, representing varied backgrounds, environmental exposures, and genetic profiles. In these trials, patients already receiving antifibrotic therapy were required to be on a stable dose for at least 12 weeks to ensure consistent baseline therapy, while those off antifibrotics for at least 8 weeks could enroll and were permitted to initiate treatment if IPF progression or acute exacerbation occurred after Week 12 [4, 10]. While our primary focus was on the 18‐mg dose, both the 9‐ and 18‐mg doses of nerandomilast were evaluated in the trials, each with a follow‐up period of 52 weeks.

In both trials, patients continued to experience significant FVC decline despite widespread use of antifibrotics, underscoring the need for additional therapies. The greater decline in FVC among patients already on nintedanib likely reflected more advanced or aggressive disease at baseline. Importantly, nerandomilast slowed disease progression in both groups, supporting its potential benefit regardless of prior antifibrotic use [4]. It, however, did not significantly impact acute exacerbations, respiratory hospitalizations, or mortality. In the FIBRONEER‐IPF trial, the efficacy of nerandomilast was reduced when combined with pirfenidone due to a drug–drug interaction that halved plasma concentrations [10]. Finally, the observed effects on patient‐reported outcomes were inconsistent and remain inconclusive in both trials [4, 10].

Adverse effects observed across all trials were generally similar between groups, with no specific side effect uniquely attributable to nerandomilast. Diarrhea was the most commonly reported event, though it was evenly distributed and likely related to concomitant nintedanib therapy. Only a small proportion of participants discontinued treatment due to diarrhea [4, 10, 15, 16]. While neither of the two large trials individually demonstrated a significant mortality benefit with nerandomilast, our pooled analysis of both trials suggested a possible improvement in survival [4, 10].

Our meta‐analysis offers several notable strengths. By applying broad inclusion criteria, we were able to compile the first and largest meta‐analysis to date on this subject. We adhered to a rigorous study protocol and, whenever feasible, restricted our analyses to the intention‐to‐treat population. Finally, all the RCTs demonstrated a low risk of bias on assessment. Despite these strengths, several limitations exist. With only four trials included, the statistical power was limited, and true effects on outcomes, such as mortality, acute exacerbations, DLCO, and adverse events, may have been missed. Although the pooled analysis demonstrated a lower risk of mortality, the individual trials were not adequately powered to assess mortality as a primary outcome. Additionally, the number of mortality events was low across studies. Therefore, this result should be interpreted with caution, as it may be subject to imprecision.

Furthermore, since the included studies covered two broad ILD populations (IPF and PPF), our results should be generalized cautiously, as differences in baseline therapy, disease characteristics, and exposures could influence outcomes. Although we have done subgroup analysis based on the types of ILD, it should be interpreted with caution because subgroup analyses are observational in nature. Potential patient overlaps between early‐phase trials (Phase Ic and Phase II), conducted within the same drug development program, cannot be entirely excluded. This could potentially overestimate the precision and magnitude of treatment effects. The lack of access to individual patient data also restricted our ability to explore key effect modifiers, such as concomitant treatments or pathogen‐specific factors.

Our study highlights several new implications for research and clinical practice. The results suggest a role as an adjunct to nintedanib, pirfenidone, or both. Future studies should focus on each subgroup individually to clarify the true effects of this agent. Studies focusing on larger, longer trials to assess mortality, exacerbation‐free survival, and hospitalizations, using time‐to‐event analyses, are warranted to ascertain its clinical benefit. We also suggest future studies stratify patients by baseline disease severity (mild, moderate, or severe) to better determine its role across different stages of fibrosis.

Nerandomilast appears to slow lung function decline in PF without added safety risks. Although a lower pooled risk of mortality was observed, individual trials were not powered for mortality outcomes, and event rates were low; therefore, this finding should be interpreted cautiously. Given the heterogeneity of PF phenotypes and trial designs, further large‐scale RCTs should explore standardized outcomes, subgroup effects, and combination strategies with nintedanib or pirfenidone.

## Author Contributions

**Humna Shahzad:** conceptualization; data curation; formal analysis; investigation; software; validation; visualization; writing – original draft. **Usama Afzaal:** conceptualization; data curation; formal analysis; investigation; methodology; software; visualization; writing – original draft. **Fahad Saleem:** data curation; investigation; methodology; software; validation; visualization; writing – original draft. **Ahmad Hassan Gul:** data curation; formal analysis; methodology; writing – original draft. **Freya Thummar:** investigation; methodology; resources; validation; visualization; writing – original draft. **Hafiz Nadir Murtaza:** investigation; validation; visualization; writing – original draft. **Abel Gelan:** investigation; methodology; resources; validation; visualization; writing – original draft. **Maria Ahsan:** data curation; formal analysis; investigation; methodology; writing – review and editing. **Rafay Irfan:** investigation; methodology; resources; validation; visualization; writing – original draft. **Ahmad Nawaz:** formal analysis; investigation; methodology; validation; visualization; writing – original draft. **Uzair Jafar:** investigation; methodology; resources; validation; visualization; writing – original draft. **Muhammad Ehsan:** data curation; formal analysis; investigation; methodology; writing – review and editing. **Asma'a Munasar Ali Alsubari:** data curation; formal analysis; investigation; methodology; writing – review and editing. **Ahmed Nadeem:** resources; supervision; validation; visualization; writing – review and editing. **Praveen Kumar Komminni:** supervision; validation; project administration; visualization; writing – review and editing. **Juan Iribarren:** investigation; methodology; project administration; supervision; validation; visualization; writing – review and editing.

## Funding

The authors have nothing to report.

## Ethics Statement

We confirm that we have read the Journal's position on issues involved in ethical publication and affirm that this report is consistent with those guidelines.

## Conflicts of Interest

The authors declare no conflicts of interest.

## Supporting information


**Figure S1:** Funnel plot for the effect of nerandomilast versus placebo on the mean change in FVC from baseline.
**Figure S2:** Funnel plot for the effect of nerandomilast versus placebo on the change from baseline in DLco percentage of the predicted value.
**Figure S3:** Funnel plot for the effect of nerandomilast versus placebo on the risk of all‐cause mortality.
**Figure S4:** Forrest plot for the effect of nerandomilast versus placebo on the risk of adverse events.
**Figure S5:** Funnel plot for the effect of nerandomilast versus placebo on the risk of adverse events.
**Figure S6:** Forrest plot for the effect of nerandomilast versus placebo on the risk of serious adverse events.
**Figure S7:** Funnel plot for the effect of nerandomilast versus placebo on the risk of serious adverse events.
**Figure S8:** Forrest plot for the effect of nerandomilast versus placebo on the risk of adverse events leading to treatment discontinuation.
**Figure S9:** Funnel plot for the effect of nerandomilast versus placebo on the risk of adverse events leading to treatment discontinuation.
**Table S1:** PRISMA checklist.
**Table S2:** Search strategy for electronic databases.

## Data Availability

Data will be available from authors on request.

## References

[1] M. Jiang , W. Bu , X. Wang , et al., “Pulmonary Fibrosis: From Mechanisms to Therapies,” Journal of Translational Medicine 23, no. 1 (2025): 515, 10.1186/s12967-025-06514-2. 40340941 PMC12063347

[2] Q. Zheng , I. A. Cox , J. A. Campbell , et al., “Mortality and Survival in Idiopathic Pulmonary Fibrosis: A Systematic Review and Meta‐Analysis,” ERJ Open Research 8, no. 1 (2022): 00591–02021, 10.1183/23120541.00591-2021. 35295232 PMC8918939

[3] H. K. Kang and J. W. Song , “Progressive Pulmonary Fibrosis: Where Are We Now?,” Tuberculosis Respiratory Disease 87, no. 2 (2024): 123–133, 10.4046/trd.2023.0119. PMC10990610 38111100

[4] T. M. Maher , S. Assassi , A. Azuma , et al., “Nerandomilast in Patients With Progressive Pulmonary Fibrosis,” New England Journal of Medicine 392 (2025): NEJMoa2503643, 10.1056/NEJMoa2503643. 40388329

[5] V. Cottin , S. Guéguen , H. Nunes , et al., “Treatment of Idiopathic Pulmonary Fibrosis With Capsule or Tablet Formulations of Pirfenidone in the Real‐Life French RaDiCo‐ILD Cohort,” Advances in Therapy 39, no. 1 (2022): 405–420, 10.1007/s12325-021-01961-x. 34757602

[6] T. E. King , W. Z. Bradford , S. Castro‐Bernardini , et al., “A Phase 3 Trial of Pirfenidone in Patients With Idiopathic Pulmonary Fibrosis,” New England Journal of Medicine 370, no. 22 (2014): 2083–2092, 10.1056/NEJMoa1402582. 24836312

[7] L. Richeldi , R. M. Bois , G. Raghu , et al., “Efficacy and Safety of Nintedanib in Idiopathic Pulmonary Fibrosis,” New England Journal of Medicine 370, no. 22 (2014): 2071–2082, 10.1056/NEJMoa1402584. 24836310

[8] F. E. Herrmann , C. Hesslinger , L. Wollin , and P. Nickolaus , “BI 1015550 Is a PDE4B Inhibitor and a Clinical Drug Candidate for the Oral Treatment of Idiopathic Pulmonary Fibrosis,” Frontiers in Pharmacology 13 (2022): 838449, 10.3389/fphar.2022.838449. 35517783 PMC9065678

[9] Y. Tagami , M. Kawai , K. Okamura , and H. Kitamura , “Oral Antifibrotic Agents for the Treatment of Idiopathic Pulmonary Fibrosis: A Systematic Review and Network Meta‐Analysis of Randomized Controlled Trials,” Journal of Clinical Question 2, no. 4 (2025), 10.69854/jcq.2025.0021.

[10] L. Richeldi , A. Azuma , V. Cottin , et al., “Nerandomilast in Patients With Idiopathic Pulmonary Fibrosis,” New England Journal of Medicine 392 (2025): NEJMoa2414108, 10.1056/NEJMoa2414108. 40387033

[11] F. Bonella , P. Spagnolo , and C. Ryerson , “Current and Future Treatment Landscape for Idiopathic Pulmonary Fibrosis,” Drugs 83, no. 17 (2023): 1581–1593, 10.1007/s40265-023-01950-0. 37882943 PMC10693523

[12] J. P. T. Higgins , J. Thomas , J. Chandler , et al., Cochrane Handbook for Systematic Reviews of Interventions (Wiley, 2019), 10.1002/9781119536604.

[13] PRISMA‐P Group , D. Moher , L. Shamseer , et al., “Preferred Reporting Items for Systematic Review and Meta‐Analysis Protocols (PRISMA‐P) 2015 Statement,” Systematic Reviews 4, no. 1 (2015): 1, 10.1186/2046-4053-4-1. 25554246 PMC4320440

[14] J. P. Higgins , J. Savović , M. J. Page , R. G. Elbers , and J. A. Sterne , “Assessing Risk of Bias in a Randomized Trial,” in Cochrane Handbook for Systematic Reviews of Interventions (John Wiley & Sons, Ltd, 2019), 205–228, 10.1002/9781119536604.ch8.

[15] L. Richeldi , A. Azuma , V. Cottin , et al., “Trial of a Preferential Phosphodiesterase 4B Inhibitor for Idiopathic Pulmonary Fibrosis,” New England Journal of Medicine 386, no. 23 (2022): 2178–2187, 10.1056/NEJMoa2201737. 35569036

[16] T. M. Maher , C. Schlecker , D. Luedtke , S. Bossert , D. F. Zoz , and A. Schultz , “Phase I Studies of BI 1015550, a Preferential Phosphodiesterase 4B Inhibitor, in Healthy Males and Patients With Idiopathic Pulmonary Fibrosis,” ERJ Open Research 8, no. 4 (2022): 00240‐02022, 10.1183/23120541.00240-2022. 36299369 PMC9589333

[17] T. H. Sisson , P. J. Christensen , Y. Muraki , et al., “Phosphodiesterase 4 Inhibition Reduces Lung Fibrosis Following Targeted Type II Alveolar Epithelial Cell Injury,” Physiological Reports 6, no. 12 (2018): e13753, 10.14814/phy2.13753. 29952109 PMC6021279

[18] E. Garbe , F. Hoti , T. Schink , et al., “Long‐Term Safety of Roflumilast in Patients With Chronic Obstructive Pulmonary Disease, a Multinational Observational Database Cohort Study,” International Journal of Chronic Obstructive Pulmonary Disease 19 (2024): 1879–1892, 10.2147/COPD.S465517. 39185393 PMC11345007

[19] D. Singh , A. Emirova , C. Francisco , D. Santoro , M. Govoni , and M. A. Nandeuil , “Efficacy and Safety of CHF6001, a Novel Inhaled PDE4 Inhibitor in COPD: The PIONEER Study,” Respiratory Research 21, no. 1 (2020): 246, 10.1186/s12931-020-01512-y. 32962709 PMC7510119

[20] B. L. Graham , V. Brusasco , F. Burgos , et al., “2017 ERS/ATS Standards for Single‐Breath Carbon Monoxide Uptake in the Lung,” European Respiratory Journal 49, no. 1 (2017): 1600016, 10.1183/13993003.00016-2016. 28049168

